# Metabolic impact of endogenously produced estrogens by adipose tissue in females and males across the lifespan

**DOI:** 10.3389/fendo.2025.1682231

**Published:** 2025-10-17

**Authors:** Angel A. Lee, Laura J. Den Hartigh

**Affiliations:** ^1^ Department of Medicine: Metabolism, Endocrinology, and Nutrition, University of Washington, Seattle, WA, United States; ^2^ UW Medicine Diabetes Institute, Seattle, WA, United States

**Keywords:** sex steroids, sex hormones, adipocyte, aromatase, estradiol, estrone, obesity, menopause

## Abstract

The aged population, expected to double by 2050, makes up a large proportion of people living with metabolic disease. Obesity rates in the elderly are rapidly increasing, with estimates that nearly 40% of men and women over the age of 60 are classified as obese. White adipose tissue (WAT) is a highly metabolically active organ that undergoes significant changes during both obesity and aging, and metabolic dysfunction in WAT is a major cause for elevated diabetes risk. A marked difference in fat distribution is often reported between men and women. Many studies suggest that pre-menopausal women are protected from the accumulation of visceral adiposity due to gonadal estrogen, which exerts cardiometabolic benefits. Men with obesity harbor a disproportionately higher volume of intra-abdominal fat than premenopausal age-matched women with obesity, an effect that is negated by menopause as women begin to gain intra-abdominal fat. Post-menopausal women are at increased risk of developing diabetes, which can be mitigated by estrogen replacement therapy, suggesting an important role for sex steroids in diabetes risk. In addition to being highly responsive to gonadal estrogens, WAT has the capacity to convert androgens into estrogens, which may similarly impact WAT distribution and metabolism. Estrogens, comprised primarily of estrone (E1) and estradiol (E2) within WAT, are biosynthesized from circulating androgens androstenedione (A4) and testosterone (T) by aromatase (CYP19A1), which is highly expressed in human and mouse adipose tissue. In post-menopausal women, WAT becomes the predominant source of estrogen production, with age-associated increases in WAT aromatase expression that are mirrored by obesity. In contrast to ovarian estrogen production, in which E2 is the predominant estrogen type, E1 tends to be the predominant estrogen post-menopause. To date, little is known about WAT-derived estrogens and their impact on metabolic health, but emerging evidence suggests that increased E1 levels may contribute to metabolic dysfunction in aging. This review will introduce known sex differences in adipose metabolism associated with aging, obesity, and diabetes, and discuss the impact of WAT-derived sex hormones on local and systemic metabolism.

## 1 Introduction

The global rise in obesity has become one of the most pressing health challenges of our time as a leading contributor to global morbidity and mortality. Between 1975 and 2014, the average body weight of adults worldwide dramatically increased, with women gaining an equivalent of an additional 1.5 kg per decade (1). The striking rise in body mass index (BMI) is a comorbidity to other metabolic diseases, including type 2 diabetes (T2D), which is driven in part by dysregulated white adipose tissue (WAT) function. WAT is a highly metabolically active organ that undergoes significant physiological changes during obesity as well as aging. WAT can expand via increased recruitment of pre-adipocytes, hence increasing the total number of adipocytes (hyperplasia), or via the enlargement of existing adipocytes (hypertrophy). WAT exists as many depots distributed throughout the body, and in general are categorized as either subcutaneous (sWAT) or visceral/omental (vWAT). sWAT is the most abundant depot in healthy people and contributes to metabolic health (2, 3). By contrast, excess vWAT is associated with metabolic syndrome, which includes the constellation of type 2 diabetes, hypertension, insulin resistance, dyslipidemia, systemic inflammation, and atherosclerosis (4, 5). During periods of nutrient excess (i.e. obesity), WAT may become severely dysfunctional due to maximization of WAT expansion potential, resulting in ectopic fat accumulation and lipotoxicity in other organs (2). In women, the transition to menopause significantly increases metabolic risk, with postmenopausal women facing a five times greater risk of central obesity compared to premenopausal women (6–8). This significant shift in fat distribution from subcutaneous regions in the hips and thighs to visceral depots is attributed to the loss of ovarian sex steroid production, in which case, adipose tissue then becomes the primary site of estrogen production (7, 9).

The gonads are classically thought to contribute to the majority of circulating sex hormone levels, and by extension are assumed to primarily determine sex steroid exposure to other tissues in the body. This largely ignores the contribution of local tissues to extra-gonadal sex hormone effects (10). The metabolic impact of estrogens produced in adipose tissue, for example, remains poorly understood, and will be a major focus of this review. During reproductive years, when gonadal estradiol (E2) is the dominant estrogen, premenopausal women tend to accumulate more subcutaneous fat, particularly in the hips and thighs (10). This pattern presents metabolic benefits, as subcutaneous fat is associated with protective effects, while visceral fat promotes a greater metabolic risk (10). However, in postmenopausal women, circulating estrogen levels decline, and the remaining levels of circulating estrogens reflect what is produced in extragonadal sites like adipose tissue (11). Studies that support circulating levels of estrogen being a secondary outcome of estrogen production for postmenopausal women and men clarify what may occur in extragonadal tissues but does not capture the local or tissue-specific actions of estrogen itself. The mechanisms and consequences underlying the production of estrogen in different adipose tissue depots remain unclear. Additionally, the metabolic impact on adipose tissue by endogenously produced estrogens appears to be influenced by age, sex, and metabolic disease. These variations in estrogen regulation and its implications for metabolic health warrant additional investigation.

This review will investigate the impact of age, sex, and metabolic disease on endogenous adipose tissue estrogen metabolism, including differences between subcutaneous and visceral fat depots. Observational studies in humans across the age span and preclinical studies in rodent models that have contributed to our knowledge regarding the relationships between adipose and sex steroids will be discussed. Firstly, adipocyte metabolism in healthy, aging, obese, and diabetic individuals will be presented, followed by the response of adipose tissue to estrogen. Next, we will explore the regional differences of estrogen activity in different depots of fat, including estrogen conversion mechanisms in adipose tissue and the changes that occur with aging. Finally, the impact of these endogenously produced estrogens on metabolic function will be discussed. This review thus offers a unique adipocentric perspective on estrogen metabolism in health and disease across the lifespan.

## 2 Adipocyte metabolism

In a healthy state, WAT importantly contributes to the maintenance of lipid and glucose homeostasis. Adipocytes store triglycerides and release free fatty acids (FFA), as well as synthesize and secrete adipokines, to maintain metabolic homeostasis. The WAT secretome is composed of cytokines, adipokines, and other factors, which can be reviewed here (12), and adipose depot location greatly influences metabolic health (13, 14). However, the differences in depot specific WAT sites and their molecular properties are much less understood (15). Adipocytes within WAT can expand in both number (hyperplasia) and in size (hypertrophy) which has been shown to be regulated by nutrient availability and sex steroids (10, 16). Healthy expansion of WAT includes increased vascularization and anti-inflammatory signals (17, 18). Thus, adipose tissue can be considered as an energy balance “hub” that integrates the body’s requirements for energy storage and utilization by other organ systems.

To understand the role of WAT on metabolic function and dysfunction, it must first be understood that while obesity is commonly defined using BMI, it is not a reliable indicator of metabolic health on its own. Subsets of individuals who classify as having obesity can have “metabolically healthy obesity’ (MHO) or “metabolically unhealthy obesity” (MUHO), and it is also possible to be “Metabolically unhealthy with a normal weight” (MUHNW) (19), suggesting there are clear exceptions that may be misclassified if one were to rely on BMI alone. Several studies have been conducted on these subsets of patients to clarify the root connection between obesity and metabolic decline. Demographics ranging between men, women, and postmenopausal women show that there is a correlation between a beneficial phenotype for metabolic outcomes and subcutaneous fat accumulation, whereas visceral fat accumulation, including omental adipocyte hypertrophy, leads to a decline in metabolic health (20–25). Adipose tissue distribution can contribute to metabolic dysfunction associated with aging, obesity, and diabetes, to be discussed in more detail in the next sections.

### 2.1 Aging

**Figure 1:**
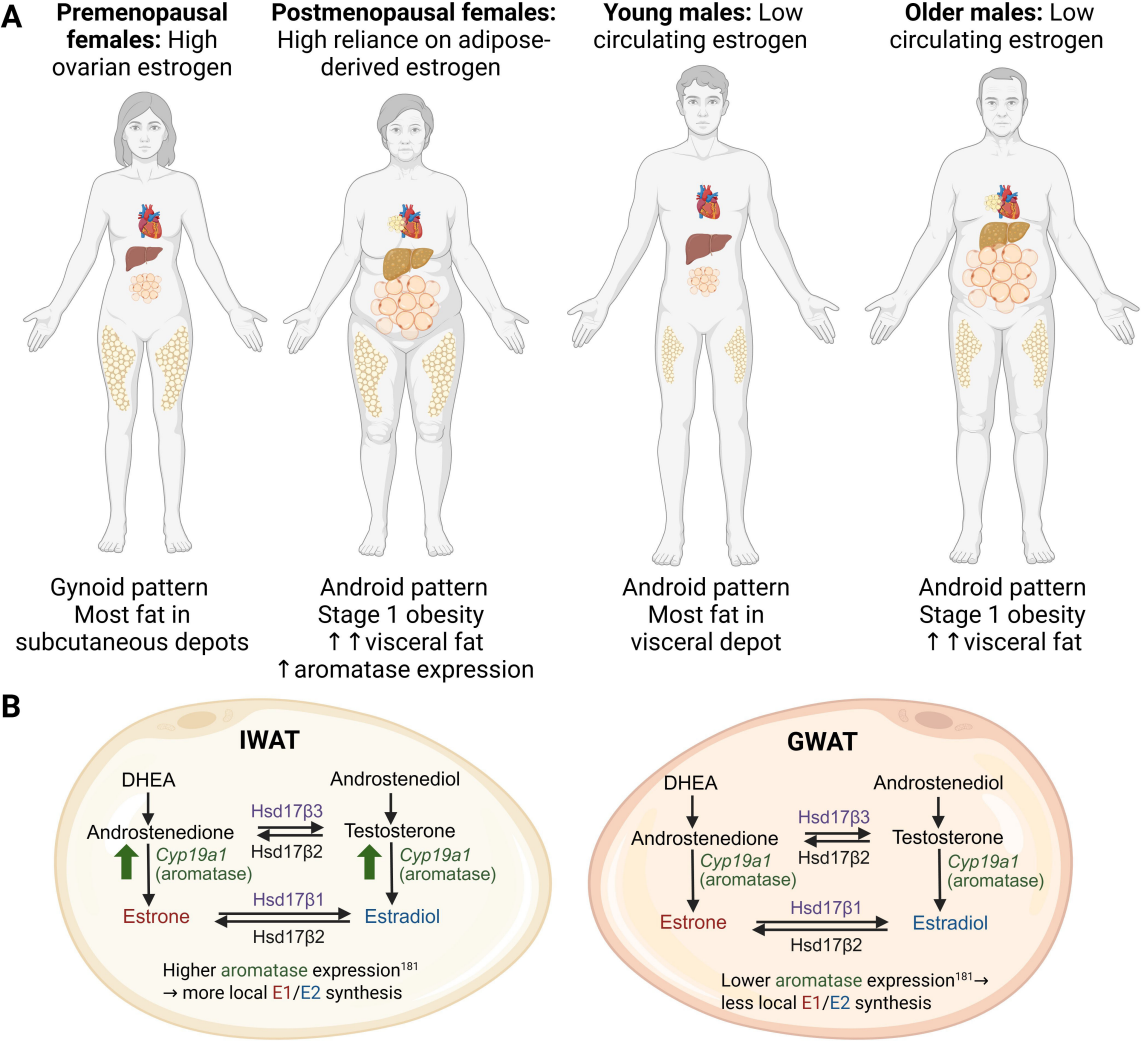
**(A)** Fat distribution changes across the age span in women and men. Premenopausal women tend to store most fat in subcutaneous depots. After menopause, more fat accumulates in the visceral compartment, including ectopic fat surrounding the heart and in the liver. Young and older men tend to accumulate most of their fat in the intra-abdominal region, with more visceral and ectopic fat accumulation as they age. **(B)** Estrogen synthesis pathways in subcutaneous inguinal white adipose tissue (IWAT) and visceral gonadal white adipose tissue (GWAT). In both IWAT and GWAT, estrogens can be converted from androstenedione and/or testosterone due to the presence of aromatase (Cyp19a1). DHEA, androstenedione, androstenediol, and testosterone enter WAT from the circulation. Estrone (E1) results from androstenedione conversion, while estradiol (E2) is a product of testosterone conversion by aromatase. E1 and E2 can interconvert due to the action of 17-beta hydroxysteroid dehydrogenases 1 and 2 (Hsd17β1 and Hsd17β2). Aromatase expression and activity tends to be higher in IWAT. Created in BioRender. Lee, **(A)** (2025) https://BioRender.com/62mlzmg.

The aged population, herein pertaining to adults over the age of 60, is expected to double by 2050 (26). Aging is a major contributor to the growing global prevalence of metabolic diseases, which include the constellation of obesity, diabetes, and cardiovascular disease (CVD). Obesity rates among the elderly are rapidly increasing, with estimates that 37.5% of men and 39.4% of women over age 60 are classified as obese in the United States (27). With aging, fat mass and tissue distribution go through significant changes. Fat depot sizes reach a peak in middle to advanced age (28), following a substantial decline, which appears to be a result of decreased hypertrophy rather than hyperplasia, as the capacity for WAT to continue preadipocyte differentiation declines with age (29–31). Included in this decline is a redistribution of fat from subcutaneous to visceral depots, particularly in post-menopausal women, which is associated with an increased risk for metabolic dysfunction, including insulin resistance (IR), as adipose tissue releases excess FFA and inflammatory mediators that impair insulin signaling (32, 33). Additionally, with age, the decline in WAT mass is associated with accumulation of fat in ectopic regions in non-adipose tissue organs like skeletal muscle (34), liver (35), and bone marrow (36), which exacerbates metabolic dysfunction (37). Aging-related changes in WAT distribution in women and men are depicted in **Figure 1**.

A relationship between aging and chronic, low-grade inflammation exists, in which a term called “inflammaging” arose, coined by Dr. Franceschi in the year 2000 (38). With this, proinflammatory cytokines are notably increased, including immune cells infiltrating WAT (38). Aged WAT is characterized by a proinflammatory microenvironment with elevated expression of inflammatory genes associated with metabolic disease (39). Aging is also associated with macrophage infiltration into primarily visceral adipose depots which drives a pro-inflammatory state and further increases adipose dysfunction (40). In terms of function and metabolism, older WAT exhibits reduced lipolysis and lipid storage capacity, driven by increased fibrosis and reduced plasticity (41). Additionally, WAT from older individuals exhibits a decline in anti-inflammatory adipokines like adiponectin, and an upregulation in pro-inflammatory cytokines that include tumor necrosis factor alpha (TNF-α) and interleukin-6 (IL-6) which can be reviewed here. In post-menopausal women, a similar pattern of decreased circulating estrogen levels has been correlated to decreased adiponectin secretion and elevated levels of TNF-α and IL-6 (42, 43). This chronic inflammation in aging WAT contributes to hepatic insulin resistance and systemic metabolic decline (44). The association between decreased adiponectin levels in postmenopausal women exacerbating metabolic dysfunction is supported by a cross-sectional study by Karim et al. which suggests an inverse association with endogenous concentrations of estrogen and adiponectin and ghrelin and a positive association with leptin and endogenous estrogens (45). While cross-sectional studies may be limited in design, a longitudinal study by Tai et al. in Taiwanese postmenopausal women showed that higher serum adiponectin levels were associated with lower BMI and decreased risk of hyperlipidemia. Similar results were seen in another cross-sectional study in which menopausal age category was accounted for, but with no influence (46). Associations between decreased adiponectin and enhanced insulin resistance and other metabolic diseases is supported by several other studies (47–49).

### 2.2 Obesity and diabetes

Obesity, which is characterized by an expansion of WAT, occurs through two primary mechanisms: hyperplasia and hypertrophy. Hyperplasia, referring to an increase in the number of adipocytes, is associated with an increase in subcutaneous fat volume and protective signals, and is associated with MHO (50). A greater number of small adipocytes has been associated with improved insulin sensitivity, reduced inflammation, and less ectopic lipid accumulation, as the production of new cells leads to a greater capacity for nutrient storage (51). On the contrary, hypertrophic adipocytes, in which existing adipocytes enlarge, is associated with increased dysfunction and MUHO (50), which contributes to IR and subsequent T2D (52, 53). The molecular and genetic mechanisms underlying obesity-driven hyperplasia and hypertrophy are reviewed here (54). Hypertrophic adipocytes are associated with an interference in lipolysis and adipokine secretion. This reduces cellular stability, increasing the risk of cell death resulting in the chronic, low-grade inflammation that we see in tandem with metabolic syndrome (25). Hypertrophy leads to multiple fold increase in infiltration of macrophages in WAT (55, 56), which promotes a pro-inflammatory and insulin resistant environment. There are several mechanisms that lead to this state, including the promotion of cell death. As WAT expands beyond its capacity, hypoxia is induced, leading to cellular stress and necrosis (57). This exacerbates macrophage inflammation, and thus the chronic pro-inflammatory state that impairs adipocyte function and insulin sensitivity which contributes to systemic dysregulation (57, 58). Furthermore, dysfunctional adipocytes caused by obesity have impaired storage capabilities, promoting the release of FFA into circulation and ectopic fat accumulation which interfere with insulin signaling pathways and contribute to systemic insulin resistance and eventual T2D (44). The pathophysiology of obesity-related diabetes has several nuances related to the disruption of metabolic homeostasis caused by increased adiposity. Obesity-induced insulin resistance is driven by adipocyte dysfunction, coupled with chronic inflammation, and ectopic lipid deposition in non-adipose tissues, similar to the effects seen in aging, which other reviews describe as a compounding effect (59, 60).

## 3 Sex steroids and metabolic disease

Sex steroid synthesis pathways have been well described (61, 62). Briefly, as shown in **Figure 1**, gonadal sex steroids are synthesized from cholesterol by the enzymatic action of steroidogenic acute regulatory protein (StAR) and CYP11A1. Cholesterol-derived pregnenolone (Preg) can be further metabolized into progesterone or dehydroepiandrosterone (DHEA) by specific enzymes, followed by androstenedione (A4) and/or testosterone (T), which can be terminally converted into estrogens. A4 is primarily converted into E1, while T is converted into E2, both directed by CYP19A1 (aromatase) activity. E1 and E2 can interconvert due to the activity of several hydroxysteroid dehydrogenase (HSD) enzymes, HSD17β subtypes 1, 7, and 12 (62). E2 derived from testosterone is the primary estrogen produced from the ovaries, while DHEA-derived A4 leads primarily to E1 in adipose tissue (63). Thus, E2 tends to dominate in people with high gonadal function, while E1 becomes more prevalent as gonadal function declines, as occurs in aging, obesity, PCOS, and post-menopause (64–66). The impact of circulating sex steroids on metabolic health and aging is summarized in **Table 1**.

**Table 1:** Summary of the impact of circulating sex steroids on metabolic disease and aging in humans.

Circulating sex steroid	Association with metabolic disease	Association with aging
SHBG	-Inverse association with intra-abdominal WAT and obesity (111, 172, 222–224). -Inverse association with T2D, mediated by visceral fat content (103, 110, 225, 226). -Decreases with weight gain (227). -Increases with weight loss in obese men and women (111, 228–232). -Promotes WAT flexibility in women, contributing to improved insulin sensitivity (233).	-Inversely associated with BMI in postmenopausal women (n=267) (171). -Increases following weight loss in post-menopausal women and older men (228, 230, 231).
DHEA	-Inversely associated with obesity in twins discordant for obesity (n=10) (223). -Inversely associated with BMI in premenopausal women with PCOS (n=136) (234). -Inversely associated with visceral fat in lean premenopausal women (n=30) (235). -Inversely associated with adiposity in premenopausal women with obesity (n=28) (224). -Inversely associated with visceral adiposity in healthy men (n=80) (175). -Inversely correlated with insulin resistance in men (236).	-Aging decreases DHEA levels in men (n=217) (237).
A4	-Inversely associated with BMI, adiposity, and abdominal adipose mass in pre-menopausal women (n=46) (238). -Inversely associated with BMI in premenopausal women with PCOS (n=136) (234). -Positively associated with obesity in premenopausal women (n=28) (224).	-Aging decreases A4 levels in men (n=217) (237).
E1	-In twin men discordant for obesity (n=18 pairs), E1 levels are higher in the heavier twin (172). -Associated with BMI and adiposity in women (170, 173, 174). -Associated with BMI and waist circumference in men (169, 175, 176). -Associated with impaired fasting glucose and insulin resistance in men (239) and women (66, 174).	-Associated with BMI in postmenopausal women (n=267) (171, 174). -Associated with lower-body obesity in women with obesity (240). -In postmenopausal women, E1 is inversely correlated with adiponectin and insulin sensitivity (n=101) (174).
E2	-Inversely associated with obesity in premenopausal women (241–244). -Associated with insulin sensitivity in premenopausal women (245, 246). -Hyperestrogenemia is associated with insulin resistance (247, 248). -In twin men discordant for obesity (n=18 pairs), E2 levels are higher in the heavier twin (172).	-Protective effect on excess adiposity of E2 replacement in post-menopausal women (n=40) (249). -In postmenopausal women, E2 is inversely correlated with adiponectin and insulin sensitivity (n=101) (174, 250). -Associated with BMI and adiposity in postmenopausal women (171, 174, 251). -Declines with aging in both men (252, 253) and women. -Lifestyle-mediated weight loss decreases E2 in older women (230). -Inversely related to cognitive decline (254).
T	-High association with WAT mass and leptin in women (170, 235, 255–257). -Lower association with BMI, WAT mass and leptin in men (169, 170, 172, 175, 255–258). -Weight loss increases T in older men, and decreases T in older women (229, 230, 259). -Protective against T2D in men (260). -Inversely correlated with insulin resistance in men (236).	-Aging decreases T levels in men (n=217) (237). -Associated with BMI in postmenopausal women (n=267) (171).

Blue text: beneficial associations with metabolic disease or aging; red text: detrimental associations with metabolic disease or aging.

A notable sexual dimorphism exists in body fat composition and adipocyte metabolism, which is influenced by sex steroids that include estrogens and androgens (51) (**Figure 1**). Women generally have higher subcutaneous fat storage capacity than men, which provides some protection against metabolic dysfunction, while men tend to store more visceral fat, putting men at greater risk for developing IR (67, 68). After menopause, women tend to store a similar amount of adipose tissue in the visceral compartment as men, implicating a lack of gonadal estrogen signaling in visceral fat accumulation and subsequent increased risk for IR (69). Indeed, subcutaneous adipose tissue expresses high levels of estrogen receptors (37). Hyperplasia and hypertrophy can occur in a depot-specific manner, as intra-abdominal fat depots primarily expand through hypertrophy, while subcutaneous WAT has been seen to expand through both methods (54). Moreover, visceral and subcutaneous WAT expansion occurs in a sexually dimorphic manner. Male mice develop diet-induced obesity primarily though visceral fat hyperplasia, while female mice do so via both visceral and subcutaneous fat hyperplasia (70, 71) with sex steroids playing a major role (70, 72). The direct impact of gonadal sex steroids on WAT function is not well understood, which prompts further investigation into the sexual dimorphism of adipocyte function and the mechanisms of gonadal steroids in these respective tissues. Moreover, T2D is more prevalent in men than women, with an estimated 13% of men and 11% of women between the ages of 20–79 classified as diabetic in 2016 (67, 73). Post-menopausal women are also at increased risk of developing diabetes, an effect that can be mitigated by estrogen replacement therapy (74), suggesting an important role for estrogens in diabetes risk (**Table 1**).

Animal studies have supported the protective impact of estrogens on metabolic disease risk (summarized in **Table 2**). Female mice and rats that have undergone ovariectomy (OVX), effectively removing all gonadal estrogens, have increased adiposity and are more prone to diet-induced obesity than sham-operated mice, with elevated adipose tissue inflammation (75–79). These effects of OVX appear to be consistent across rodent species (mice and rats), strain (rats: Sprague-Dawley, Wistar), and with age at OVX (4–10 weeks of age in mice). Estrogen treatment promotes anti-inflammatory and insulin sensitizing effects in both male and female mice (80, 81), and estrogen replacement reverses some of the detrimental metabolic effects of ovariectomy (80, 82–84). By contrast, castrated male rodents have been shown to exhibit improved glucose and insulin tolerance with reduced adiposity in some studies (85, 86), and in others display worsened adiposity, WAT inflammation, and glucose tolerance (87, 88). Perturbation of estrogen signaling in mice has primarily been achieved by genetic manipulation of estrogen receptors (ERα, ERβ) or aromatase in mice. Global deletion of ERα has been shown to increase adiposity, systemic and adipose tissue inflammation, and insulin resistance in mice (89–92). Similarly, adipocyte-specific deletion of ERα increases inflammation concurrently with adipocyte hypertrophy (93). Studies of mice with global aromatase deficiency consistently show an increased propensity towards obesity and insulin resistance (94–96), and mice with adipocyte-specific aromatase overexpression exhibit improved insulin sensitivity and reduced inflammation (97). Collectively, studies in mice highlight the distinct metabolic impacts of the loss of gonadal androgens and estrogens on systemic metabolism.

**Table 2:** Summary of the impact of perturbing circulating and WAT-derived sex steroids on metabolic disease and aging in mouse models.

Mouse models	Impact on metabolism
Genetic SHBG perturbation	-Male mice overexpressing SHGB is protective against diet-induced obesity and insulin resistance (109). -No metabolic impact on SHGB overexpression in male and female mice fed a HFD (108).
Genetic estrogen signaling perturbation	-ERα KO mice have impaired insulin sensitivity and are more prone to excess adiposity (89–92, 143, 144). -Hepatic overexpression of estrogen sulfotransferase (EST), which increases the inactivation of estrogens, promotes dysregulated glucose metabolism. Conversely, loss of EST improved metabolic function in females, but worsened metabolic function in males (261). -Adipocyte-specific deletion of ERα increases adipocyte hypertrophy and inflammation (93). -Mice with aromatase deficiency are more prone to obesity and insulin resistance (94–96). -Mice with adipocyte-aromatase overexpression have improved insulin sensitivity and reduced inflammation (97).
Surgical estrogen deprivation	-Ovariectomy (OVX) increases body weight and body fat in female mice (76, 83, 84, 262–264). -OVX improves HFD-induced insulin resistance in female mice (75). -OVX of ERα mice led to decreased body weight vs. sham-operated ERα mice (91). -Castration indirectly reduces estrogen in male mice, leading to enhanced adiposity and pronounced diet-induced obesity (72).
Estrogen treatment	-E2 given to female mice attenuated high fat diet-induced weight gain and visceral adiposity (81). -E2 given to males prevented high fat diet-induced weight gain and improved glucose tolerance (265, 266). -E2 given to both male and female diet-induced obese mice led to improved WAT inflammation and insulin sensitivity (80).

In addition to being a major target of gonadal sex hormones, adipose tissue can also synthesize and store them (**Figure 1**). The decline in gonadal sex hormone production that occurs in post-menopausal women and in aged men coincides with increased sex hormone synthesis within adipose tissue (76, 98). Increased synthesis of adipose tissue-derived sex hormones has also been reported in the setting of obesity (99). The mechanisms by which adipose tissue can become a source for sex hormones will be discussed in Section 3.2.4., and emerging knowledge regarding the metabolic impact of WAT-derived sex hormones is summarized in **Table 3**.

**Table 3:** Summary of the impact of WAT-derived sex steroids on metabolic disease and aging in humans.

WAT sex steroid	Association with metabolic disease	Association with aging
A4	-Increased capacity of WAT to convert T → A4 associated with BMI (267, 268).	
E1	-Increased aromatization of A4 into E1 is associated with adiposity in healthy women (269, 270). -Increased WAT E1 content in men with obesity (139). -WAT E1 levels reportedly 5X higher than circulating levels in premenopausal women (192). -WAT E1 correlates with waist circumference in premenopausal women (192).	-Aromatization rate of A4 → E1 increases with age in women, regardless of BMI (271). -Shift towards WAT-derived E1 post-menopause increases visceral fat (62, 270). -WAT aromatase expression increases with age (272).
E2	-Increased aromatization of T into E2 is associated with adiposity in healthy women (269). -Increased WAT E2 content in men with obesity (139).	-Shift towards WAT-derived E2 post-menopause increases visceral fat (62).
T	-Low T levels may contribute to more severe adipose-insulin resistance in obese men (273). -T content is higher in WAT from obese subjects than lean (274). -Positive association between WAT T production and central obesity (63, 268, 275).	

Blue text: beneficial associations with metabolic disease or aging; red text: detrimental associations with metabolic disease or aging.

### 3.1 Sex hormone-binding globulin and adipose tissue

Sex hormone-binding globulin (SHBG) is a glycoprotein primarily synthesized in the liver. SHBG regulates the bioavailability of sex steroids in the bloodstream, including testosterone (T) and estradiol (E2). There is compelling evidence that supports SHBG’s involvement in glucose and lipid metabolism and its role as a biomarker for obesity-related disorders including T2D (100–102). The mechanisms of SHBG’s regulation in the context of sex differences and adiposity are incompletely understood. This section will discuss the current knowledge and gaps thereof.

The most established link between SHBG and metabolic health is its inverse association with insulin resistance. Low circulating SHBG levels are predictive of T2D development, independent of BMI (103). Particular single nucleotide polymorphisms (SNPs) for SHBG confer increased risk for T2D (102, 104). This brings to question if SHBG may mediate a relationship between adiposity and impaired glucose metabolism (100). Mechanistically, this seems to involve insulin’s inhibitory effect on hepatic SHBG production. Hyperinsulinemia, which is a hallmark of insulin resistance, suppresses SHBG synthesis, creating a cycle of increasing free sex steroid concentrations and worsening metabolic outcomes (105–107). Interestingly, recent studies suggest that SHBG may partially explain sex differences in glucose regulation, reporting that SHBG mediates a proportion of the association between sex and fasting glucose levels, as well as T2D incidence (101). The degree to which SHBG independently influences metabolic health, or rather if it acts as a marker of other underlying processes, is not well agreed upon in current literature. A study by Sofer et al. (2018) tested the hypothesis that SHBG provides metabolic protection by feeding transgenic mice expressing human SHBG a high-fat diet (HFD) for 4.5 months. Their results revealed no protective effects from SHBG expression on obesity or dysglycemia in either male or female mice (108). SHBG transgenic mice appeared to gain weight similarly to wild-type (WT) controls. Furthermore, fasting glucose, insulin, and insulin resistance measured using HOMA-IR found no significant differences (108). It was also observed that female SHBG transgenic mice showed higher fasting glucose levels than WT controls, suggesting that SHBG may play a detrimental role in certain contexts (108). The authors speculate that the absence of metabolic protection may result from the longer duration of the HFD in their model, which may have allowed for compensatory mechanisms to override an early protective benefit that SHBG may provide. They also acknowledge that SHBG may not be causally protective but rather serve as a biomarker for metabolic health. Contrastingly, a study by Saez-Lopez et al. (2020) reported that SHBG overexpression protected male, transgenic mice against HFD-induced obesity as well as metabolic disease. Over the course of 8 weeks, compared to WT controls on the same diet, SHBG transgenic mice demonstrated significantly less weight gain, smaller epididymal white adipose tissue (EWAT) depots, and a healthier metabolic profile including lower insulin, leptin, and resistin while also demonstrating higher adiponectin levels (109). The authors proposed that this protective effect was mediated through enhanced lipolysis in WAT, as SHBG transgenic mice had elevated expression of lipolytic genes including beta-3- adrenergic receptor (*Adrb3*), interferon regulatory factor-4 (*Irf4*), and perilipin-1 (*Plin*) and increased phosphorylation of protein kinase A (PKA), extracellular signal-regulated kinases 1/2 (ERK-1/2), and hormone sensitive lipase (HSL), which may suggest that SHBG plays a more active role in adipocyte metabolism than previously thought. A notable difference between the two studies is their duration of HFD exposure. Sofer et al. used a longer, 4.5-month model which may have allowed for metabolic adaptations, or saturation effects of any protective role SHBG may play. The shorter 8-week model by Saez-Lopez et al. may reflect a more acute metabolic response. Further support for SHBG’s protective role comes from a longitudinal human study observing changes in SHBG and diabetes risk over the menopause transition (110). This study found that increasing levels of SHBG were associated with a decreased risk of T2D after adjusting for covariates. Furthermore, stable or increasing rates of change in SHBG were independently associated with a lower risk of diabetes compared to decreasing rates of change (110). This may suggest that SHBG exerts effects on glucose regulation beyond its known role as a regulator of sex steroids. Comparisons between its role with circulating sex hormones and those that are endogenously produced are also not fully understood, especially in the context of obesity related adipocyte dysfunction.

A critical question remains: Is SHBG simply a biomarker of obesity and metabolic health, or does it act as an active contributor to metabolic regulation? Cross-sectional human studies show that low SHBG levels predict T2D and metabolic syndrome incidence, even after adjusting for adiposity (111). However, these associations may not imply causation. Studies have demonstrated that SHBG levels are inversely correlated with markers of adiposity that include BMI and WHR (111, 112). In post-menopausal women, it appears that lower SHBG concentrations are associated with higher central adiposity (113). A separate study found that SHBG levels tend to increase with age linearly in healthy post-menopausal women (114). while another suggests this increase relates to the increase of circulating free testosterone in late-postmenopausal women (115). Further studies could be beneficial to clarify the interaction between hormonal aging and metabolic health in postmenopausal women with and without metabolic syndrome. Despite numerous studies that show a relationship between SHBG and adiposity, the findings are not uniform across all populations. A 1997 study found that SHBG negatively correlated with BMI, WHR, insulin, and testosterone levels in both premenopausal and postmenopausal women, however, E2 levels correlated positively with SHBG only in the premenopausal group (116). The relationship between SHBG and IAAT similarly does not reach a consensus across studies. Several cross-sectional studies have shown an inverse relationship between SHBG and IAAT, suggesting that visceral fat may actively suppress SHBG synthesis, potentially through inflammatory pathways or hepatic fat accumulation (111, 117). Contrasting to these findings, the first of a kind to publish longitudinal data regarding the relationship of IAAT gain and SHBG from postmenopausal women indicate that higher baseline SHBG levels may predict greater IAAT gain over time, a finding that challenges previous cross-sectional studies (118). These inconsistencies call for additional investigation into the temporality and causality of the SHBG-adiposity relationship.

### 3.2 Estradiol and estrone in metabolism

Estrogens influence many physiological processes including lipid metabolism and adipose tissue distribution. Estrogen has three primary forms, estrone (E1), estradiol (E2), and estriol (E3). Three estrogen receptors have been described: ERα, ERβ, and G-protein coupled estrogen receptor 1 (GPER1), also known as G-protein coupled receptor 30 (GPR30). Upon activation, estrogen receptors translocate to the nucleus where they dimerize and bind to specific DNA sequences, termed estrogen response elements, to initiate estrogen-dependent gene transcription. Estrogens can also exert receptor-mediated non-genomic effects via GPER1, including interactions with cell membrane-associated complexes such as caveolin-1, other G-proteins, and receptor tyrosine kinases such as EGFR, IGF-1, and MAPK (119, 120). E2 has the highest affinity for ERα and ERβ and is thus considered the strongest estrogen (121). E1 and E3 are weaker estrogens, with higher affinity for ERα and ERβ, respectively (121). Only E1 and E2 have reported agonism for GPER1 (122). ERα and ERβ are widely distributed throughout the body, with major and roughly equivalent expression in the brain and liver (123). Notably, metabolic tissues such as WAT, skeletal muscle, bone, and the heart have higher expression of ERα, while other tissues such as bone, prostate, testes, and ovaries have higher expression of ERβ. To date, GPER1 expression has been reported in reproductive tissues (testes, prostate, and endometrium), immune cells, metabolic tissues (adipose, liver, pancreas, and skeletal muscle), and in certain cancers (prostate, ovarian, cervical, breast, and lung) (122, 124–126). E2, or 17β-estradiol, is the predominant estrogen in premenopausal women, and it is the more biologically active form as it has a higher affinity for estrogen receptors than other estrogen forms during reproductive years (121). However, post-menopause, E1 becomes the dominat estrogen, where it is synthesized in WAT via aromatase activity (62, 127, 128). Many studies have shown that E2 beneficially influences metabolism through mechanisms involving reduced food intake and increased energy expenditure (75, 129–131). Many of the beneficial effects of E2 on energy metabolism are increasingly being attributed to its interaction with GPER1, including its rapid effects on insulin sensitivity, hepatic glucose and lipid metabolism (132, 133). However, much less is known regarding how the prevalence of E1 in postmenopausal women may influence metabolic health. Emerging evidence suggests that E1 is not associated with the same beneficial effects on insulin sensitivity reported for E2 and may promote inflammation (99, 134). Obese mice supplemented with E1 display a robust pro-inflammatory phenotype, while E2 treatment resulted in an anti-inflammatory phenotype, an effect dependent on NFκB activation by E1 that was replicated in cultured adipocytes and breast cancer cell lines (134). In addition, obesity is associated with higher circulating and WAT-derived E1 levels (64, 135–138), which may be due to the capacity for higher fat content to convert androgens into more estrogens (139, 140), and in particular E1 (62, 141). Rats fed an E1-enriched diet gained twice as much body weight as control rats (142), suggesting that E1 could promote obesity. More research is needed to improve our understanding of the metabolic impact of changing estrogens concentration and type on metabolism post-menopause.

The metabolic benefits of E2 have been well studied, with evidence that it promotes insulin-sensitivity and improved lipid metabolism in tandem with estrogen receptors, primarily ERα (90) and GPER1 (133). In mice, estrogens act via ERα and GPER1 to regulate insulin sensitivity (143). Studies have shown that ERα knockout (KO) mice in both males and females have worse metabolic profiles including developing obesity and worsened glucose homeostasis (90, 143–145). Recent research has also suggested that estrogen’s metabolic effects are not only mediated through direct action on adipocytes, but also through endothelial mechanisms of ERα that improve insulin transport to skeletal muscle (146). ERα appears to be required to induce the positive effect that E2 has on insulin sensitivity in mice (89, 90). Recent mouse models of global GPER1 deficiency report similar phenotypes. GPER1 KO mice are more prone to obesity, insulin resistance, inflammation, and dyslipidemia than control mice (147–149). Furthermore, in humans, individuals with T2D and poorly managed glucose control showed a significant decrease in *ERα* mRNA expression (145). The systemic effects of estrogens are well documented, especially regarding the role of E2 in promoting insulin sensitivity and reducing lipid accumulation, but a major limitation in our current understanding of how estrogens impact metabolism is limited by a lack of understanding the tissue-specific mechanisms that drive metabolic regulation and WAT distribution (130). Tissue-specific estrogen actions will be discussed in the next sections.

#### 3.2.1 Skeletal muscle

Current literature supports that the expression of glucose transporter-4 (GLUT4) in myocytes may be influenced by ERα in mouse models, suggesting ERα positively regulates GLUT4 expression, contributing to the systemic differences in insulin resistance seen in ERα KO mice (150). An *in vivo* experiment similarly showed that increased ERα expression increased skeletal muscle glucose uptake in ovariectomized female mice given E2 (151). Another study found that the expression of ERα in skeletal muscle had a significant, inverse relationship with adiposity and fasting insulin levels (152). However, a separate study showed that ERα is sufficient, but is not required, to protect mice from metabolic dysfunction in skeletal muscle and in women with insulin resistance and obesity (153). This necessitates future studies to consider the role of estrogens in skeletal muscle, and what mechanisms compensate for individuals with low to no ERα expression resulting from changes like aging and obesity (153). The expression of ERα is not uniform across all tissues in the body but is highly expressed in female reproductive tissues including ovaries and breast, WAT, liver, and other tissues which can be reviewed here (154). ERβ, which was not experimentally shown to regulate GLUT4 expression (150), is primarily expressed in male reproductive organs and other tissues (154). The sexual dimorphism of estrogen synthesis and estrogen receptors would benefit from additional investigations that consider the tissue-specific mechanisms in males and females and how this differs regarding our understanding of how E2 maintains a protective effect.

#### 3.2.2 Liver

There is strong evidence that estrogens regulate hepatic glucose and lipid metabolism through ERs, particularly ERα (155–157). Loss of ERα in hepatocytes appears to impair glucose tolerance and increase lipid accumulation in the liver, worsening adiposity and IR in both male and female mice, with one study reporting a greater effect in female mice (158, 159). Orally ingested estrogens have also been reported to induce acute cholestasis, causing a decrease in bile flux which is detrimental to cholesterol homeostasis (159). The role of E2 has been well established to play a regulatory metabolic role via ERα in peripheral tissues such as skeletal muscle and WAT (130, 160–162) (further discussed in Section 3.2.3). There is evidence that ERα plays a similar role in the liver, with lower, but similar expression patterns to WAT (130, 162). E2/ERα signaling appears to increase lipid and glucose metabolism in the liver by influencing transcriptional factors that increase lipolysis while decreasing lipogenesis and gluconeogenesis (162). The role of E1 in the liver is much less clear. Some studies report that elevated E1 levels may contribute to hepatic IR and increased inflammation (134). An interesting direction from studies on male patients with hepatic cirrhosis states that E1, not E2, may play a larger role on sustaining increased circulating levels of estrogen for patients with liver cirrhosis through the peripheral conversion of androgens to E1 (163, 164). This specific hormonal profile may have clinical relevance on the progression of cirrhosis and altered fat distribution. Further research regarding the conversion mechanisms of androgens to estrogens and the use of aromatase inhibitors could improve our current understanding of E1 function in the liver and how it may impact metabolic homeostasis.

#### 3.2.3 Adipose tissue

In premenopausal women, E2 promotes lipid storage predominantly in subcutaneous adipose tissue (SAT) and inhibits excessive lipid accumulation in visceral adipose tissue (VAT) (165). E2 contributes to metabolic homeostasis in adipose tissue specifically by decreasing the activity of lipoprotein lipase and subsequent lipogenesis, and has proven to influence hyperplasia of subcutaneous adipocytes (166). E2 also increases preadipocyte proliferation, suggesting an adipogenic effect (167). This protective effect appears to diminish in postmenopausal women, where a decrease in circulating E2 by the gonads leads to a deficiency, which has been associated with increased VAT deposition (168).

E1 has consistently been found to positively associate with BMI, waist circumference, and adiposity in men and women across the life span (169–176). The dominant estrogen post-menopause is E1, and while there is compelling speculation regarding its metabolic impact, there is not yet sufficient evidence for conclusive roles for E1 in energy metabolism. E2 has been studied for its endogenous metabolic benefits, yet the role of E1 in WAT function and metabolism remains much less understood. As the predominant estrogen circulating post-menopause, E1 is primarily synthesized in adipose tissue through the aromatization of androgens via aromatase (CYP19A1) (62, 177). A seminal publication suggested that E1 may exert metabolic effects distinct from those of E2, indicating that elevated E1 levels correlate with increased adiposity and insulin resistance in postmenopausal women (134). This supports the notion that E1 may contribute to metabolic dysfunction in WAT, while also supporting that E2 does not appear to have the same negative effects. It is not clear if the metabolic impact of E1 is dependent on local conversion mechanisms to E2, and if estrogen receptors and aromatase activity differ in varying depots and how this may impact the effects of E1 on metabolism. Whether E1 possesses direct metabolic effects remains a topic of interest.

The protective role attributed to E2 may be depot-specific, as ERα must be available for E2 to have a protective effect (178). Similar to what has been observed in skeletal muscle, it appears that the metabolic benefit of E2 is regulated by receptor signaling, which may vary in different fat depots. In adipose tissue, deletion of ERα (but not ERβ) is associated with metabolic decline (93, 179). In overweight pre-menopausal women, SAT in the abdominal region contains more ERα relative to ERβ than in gluteal SAT (180). Another study showed that E2/ER signaling plays a significant role in mediating sex differences of VAT accumulation, where males express less ERα in VAT than females (181).

Much has been learned about peripheral estrogen receptor-mediated effects using genetically perturbed mice. Male and female mice deficient in ERα have a higher degree of adiposity, insulin resistance, and glucose intolerance than WT mice (90, 92), a phenotype that closely resembles humans lacking either ERα or aromatase. Additional approaches to silencing ERα, including adeno-associated viral techniques and ERα^lox/lox^/Adiponectin-Cre (Adipo-ERα) mice, revealed that disrupting adipose tissue ERα recapitulated this phenotype (93), suggesting that the adipocyte is a major target of estrogens to impart beneficial effects on systemic metabolism.

#### 3.2.4 Aromatase activity and estrogen conversion in adipose tissue

**Figure 2:**
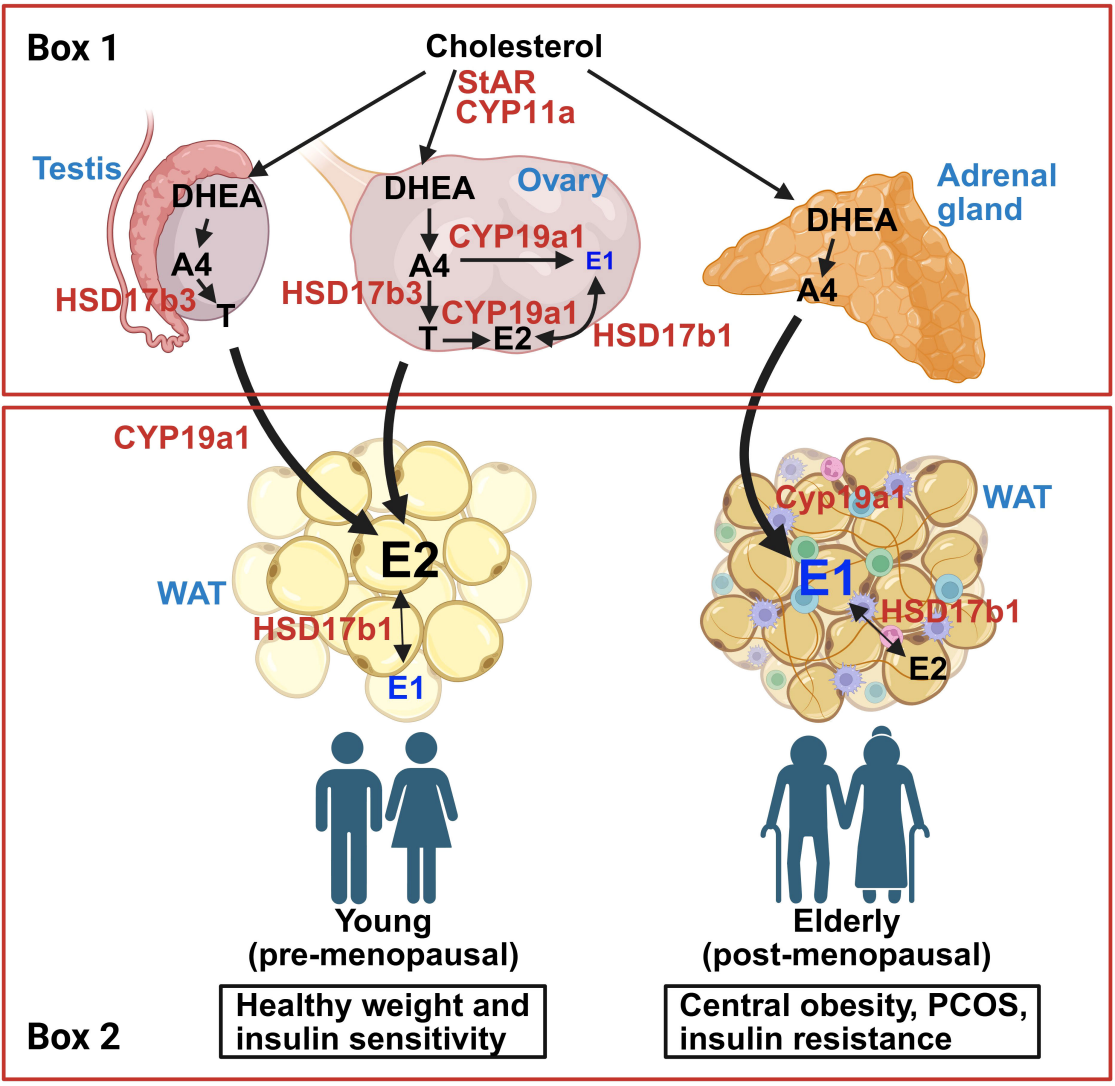
Schematic for estrogen synthesis pathways derived from cholesterol involving gonads (testes and ovaries), adrenal gland, and adipose tissue. Box 1: Gonadal and adrenal sex hormone synthesis pathways. Circulating cholesterol is transported across the mitochondrial membrane by steroidogenic acute regulatory protein (StAR) and CYP11A1 and is eventually converted to dehydroepiandrosterone (DHEA) via CYP17a1. In both testes and ovaries, DHEA is converted to androstenedione (A4) and testosterone (T) by HSD3b1 and HSD17b3, respectively. When aromatase (CYP19a1) is present, A4 and T are converted into estrone (E1) and estradiol (E2), respectively. Functional gonads release high levels of T from testes, and E2 from ovaries. Like the gonads, the adrenal gland can convert circulating cholesterol to androgens, primarily A4. Box 2: Adipose tissue androgen conversion pathways from healthy young people (left) and those with obesity, insulin resistance, PCOS, and/or advanced age (right). In people with robust gonadal function, circulating androgens (A4, T) are converted to E2 due to aromatase activity in white adipose tissue (WAT). Some E2 will convert to E1 due to HSD17b1 activity. In people with reduced gonadal function (i.e. advanced age, obesity, PCOS, and/or insulin resistance), the major circulating androgen is A4, which predominantly converts to E1 in WAT. Created in BioRender. Den Hartigh, L. (2025) https://BioRender.com/oqv7exy.

In addition to its capacity to respond to gonadal estrogens, WAT has the capacity to convert circulating androgens into estrogens (127, 128, 182) (**Figures 1** and **2**). Such endogenously converted estrogens can then function in an autocrine or paracrine manner (183, 184). Estrogen biosynthesis is catalyzed by aromatase P450, encoded by the CYP19A1 gene, which is highly expressed in human and mouse WAT (185, 186). In ovaries, aromatase is largely driven by the cyclic AMP response element binding protein (CREB) promoter (98). In contrast to the ovaries, in which the major substrate for estrogen aromatization is testosterone, the major substrates for aromatase-mediated estrogenesis in WAT are DHEA and androstenedione, and may be driven by inflammatory cytokines (62, 98). Another potential direction with promise lies in exploring the less understood pathways of aromatase activity and estrogen conversion between E2 and E1, and how it may modulate a tissue-specific outcome. WAT converts androgens to E1 via CYP19A1 (62, 141), an enzyme that appears to increase with adiposity in males due to the decline in available testosterone (187). Indeed, people with obesity have been reported to have increased CYP19A1 expression in WAT (99, 141, 188, 189). In another study, aromatase gene expression in SAT positively correlated with increased adiposity and IR, but interestingly not with circulating E2 (190). SAT tends to express aromatase in higher levels compared to VAT, whereas VAT appears to express more of 17β-hydroxysteroid dehydrogenase (17β-HSD), an enzyme which interconverts E1 and E2 (191). Another study found that, in women, aromatase levels in VAT positively correlated with adipocyte hypertrophy, suggesting that aromatase activity may be associated with VAT gain and overall metabolic dysfunction (189). These findings suggest that aromatase activity appears to regionally differ between fat depots (**Figure 1B**). Future analysis into aromatase expression levels in different depots to analyze its metabolic impact would benefit from an added component of comparing levels of E1 and E2 produced in these sites. A separate study found that E1 is the dominating form of estrogen in WAT for premenopausal women, showing a 5–10 times greater concentration of E1 in SAT and VAT compared to serum levels (192). They also found that the expression of aromatase positively correlated with the E1 concentration in VAT (192). In another study, women with obesity appear to express much higher levels of aromatase in SAT compared to VAT (193). The observed correlation between aromatase expression and its effect on adiposity and IR implicates E1 in metabolic dysfunction. Further research should seek to distinguish if E1 has direct metabolic effects or if its metabolic impact is mediated through the local conversion to E2.

Mechanistic studies in mice provide clues regarding the metabolic effects of local estrogens. Mice globally deficient in aromatase (Cyp19a1 KO) are more prone to aging-associated increase in abdominal obesity and insulin resistance (94–96). Providing exogenous E2 rescues the obesogenic phenotype in Cyp19a1 KO mice, suggesting the lack of E2 drives the increased adiposity. While rare, aromatase deficiency in humans also leads to insulin resistance and T2D (194), suggesting a beneficial effect of aromatase. In support of this, a single study has shown improved insulin sensitivity in male mice with transgenic aromatase overexpression specifically from WAT, driven largely by increased E2 (97). While these genetic studies offer a starting point in modulating estrogen production capacity in general terms, only one study has addressed the potentially divergent effects of perturbing *particular* estrogens. Qureshi et al. found that in obese mice, E1 promoted a pro-inflammatory phenotype, while E2 dampened inflammation, an effect supported by transcriptomics (134). Further, higher E1:E2 ratios were predictive of ER-positive tumor growth (134), suggesting differential impact of E1 and E2 on obesity-related breast cancer incidence. The impact of particular estrogens on breast tissue and tumor burden will be discussed in the next section.

#### 3.2.5 Breast tissue

Breast tissue appears to have a distinct estrogenic profile than other fat depots, despite breast tissue having a significant portion of WAT. Similar to WAT, the expression of aromatase in breast tissue is highly regulated and involved in estrogen synthesis. E2 in breast tissue is not associated with protective metabolic effects, but rather has been linked to proliferative tumor growth (195). Elevated aromatase expression in breast WAT increases local E2 production, which may contribute to estrogen receptor-positive (ER+) breast cancer pathogenesis (135, 195, 196). This localized estrogen synthesis differs from systemic estrogen metabolism as breast WAT appears to maintain high estrogenic activity, especially for postmenopausal women where WAT is the key source of estrogen synthesis. Moreover, tumor-bearing breast tissue has been shown to have higher aromatase expression within adipose in close proximity to the tumor than in distal tissue within the same breast (197), suggesting an adverse role for endogenously produced estrogens in breast cancer. Inflammatory cytokines secreted by hypertrophic breast tissue in individuals with obesity appear to exacerbate aromatase production and thus local estrogen production (198). In addition, the impact of post-menopausal estrogens derived from WAT on breast cancer incidence has been studied extensively, with evidence that ER-positive breast cancer incidence increases with age (199). Multivariate analyses suggest that estrogens are the most important factors associated with the elevated breast cancer risk in postmenopausal women with obesity (200). Finally, it has recently been reported that E1 and E2 derived from breast WAT have opposing pro- and anti-inflammatory transcriptional profiles, respectively (134).

### 3.4 Menopausal hormone therapy

The use of menopausal hormone therapy (MHT) to reduce metabolic dysfunction associated with menopause has been studied. However a consensus regarding its efficacy is far from established. Studies generally show that estrogen replacement therapy in postmenopausal women can promote weight loss and improve metabolic markers including fat distribution, notably by increasing subcutaneous fat and reducing visceral fat mass (201–203). The majority of MHT trials have been done in women of normal weight (BMI<30); as such, the reported impact of MHT on body weight and adiposity has been modest (74, 204–206). After adjusting for age and BMI, the Nurse’s Health Study (n=21,028) showed that MHT (estrogen alone, progesterone alone, or the combination) users had a 20% reduced risk for diabetes than non-users (207). Similarly, MHT users in the postmenopausal estrogen/progestin interventions (PEPI) trial showed improved fasting glucose and insulin levels (204). MHT has also been shown to reduce homeostatic model assessment of insulin resistance (HOMA-IR) in postmenopausal women, and with a nearly 3-fold greater benefit in postmenopausal women with T2D (204, 208). All the MHT studies listed to this point used either conjugated estrogen (CE) alone or in combination with a progestin, delivered orally. More recently, bioidentical E2 with or without a progestin has become a more common method of MHT. To date, there is very little known about the impact of other MHT delivery methods, including transdermal patches, vaginal rings, gels, creams, or suppositories, on general metabolic health. No randomized controlled trials have directly examined the metabolic impact of oral vs. transdermal HRT, but observational studies reveal the potential for reduced risk for thromboembolism and dyslipidemia with transdermal delivery when compared to oral (209, 210). This could be due to the first-pass through the liver with oral MHT, enabling increased triglycerides, coagulation factors, and C-reactive protein, which is minimized with transdermal therapy (211, 212). By contrast, while both transdermal and oral MHT delivery methods have been shown to reduce diabetes risk, oral MHT led to a greater risk reduction (213).

Conflicting data suggest that estrogen’s metabolic effects may depend on context. Initial results from the Women’s Health Initiative (WHI) showed that conjugated equine estrogen with or without medroxyprogesterone acetate let to worse cardiometabolic outcomes in postmenopausal women, dampening enthusiasm for MHT (214). It has since been suggested that, since the MHT study population in the WHI was on average more than 10 years post-menopause, metabolic benefits of MHT may be greater in women within 10 years of menopause, introducing the importance of MHT timing into the equation (215). Indeed, subsequent re-analyses of the WHI data revealed cardiometabolic benefits of MHT in women aged 50-59 (216–218). Some studies report that high circulating E2 levels are associated with increased inflammation and adipocyte dysfunction, particularly in postmenopausal individuals with obesity (174, 219). This discrepancy may be attributed to differences in estrogen metabolism, receptor expression, or interactions with other hormones such as androgens and insulin which can be reviewed here (220). Other studies support that E2’s protective effects on WAT expansion may be depot-specific, as the expression of estrogen receptor ERα, which has been shown to be necessary for E2 to maintain its protective effect of inflammation in both males and females, may be higher in subcutaneous fat (181, 221). Moreover, subcutaneous WAT has been shown to have a higher E2 conversion rate than visceral WAT (64, 192). By contrast, visceral WAT has been reported to produce more E1 than subcutaneous WAT (64). To date, it is not known how MHT impacts WAT responsiveness or estrogen conversion capacity in postmenopausal women.

## 4 Conclusions

Endogenously produced estrogens in WAT appear to impact energy homeostasis, fat distribution, and inflammation. The patterns which emerge in studies to date show that the impact of estrogens synthesized locally in WAT depots are dependent on factors like age, sex, and depot, with subcutaneous fat generally presenting higher estrogenic activity than visceral fat.

As women enter menopause, the shift in estrogen production localized to estrogens produced in WAT is primarily driven by aromatase activity. The interconversion between estradiol to estrone in WAT may play a significant role in estrone dominance over estradiol in circulation post-menopause. How estrone may influence visceral adiposity, inflammation, and insulin resistance is a topic of growing interest that requires investigation that separate the longitudinal and systemic impact presented in covariates like sex, age, depot, species, and tissue type. Examples include the difference in bioavailability and expression of key enzymes involved in estrogen production, conversion, transport, and signaling, which include but are not limited to aromatase, HSD17β1, SHBG, TNF-α, IL-6 and ERα. Further investigation to clarify how WAT responds to estrogenic signaling across the life span will lead to a more comprehensive understanding of metabolic decline with age, especially for women post-menopause. Developing therapies require the precision of understanding the interplay between the human hormonal milieu and metabolic health to manage metabolic dysfunction in diverse populations.

## Glossary

ADRB3: beta-3- adrenergic receptor
BMI: body mass index
CE: conjugated estrogen
CVD: cardiovascular disease
CYP19A1: aromatase
DHEA: dehydroepiandrosterone
E1: estrone
E2: estradiol
E3: estriol
ERα: estrogen receptor alpha
ERβ: estrogen receptor beta
ERK1/2: extracellular signal-regulated kinases 1/2
EWAT: epididymal white adipose tissue
FFA: free fatty acids
GLUT4: glucose transporter 4
GPER1: G-protein coupled estrogen receptor
GPR30: G-protein coupled receptor 30
HFD: high fat diet
HOMA-IR: homeostatic model assessment of insulin resistance
HSD: hydroxysteroid dehydrogenase
HSL: hormone sensitive lipase
IAAT: intra-abdominal adipose tissue
IL-6: interleukin-6
IR: insulin resistance
IRF4: interferon regulatory factor-4
KO: knock out
MHO: metabolically healthy obesity
MHT: menopausal hormone therapy
MUHNW: metabolically unhealthy normal weight
MUHO: metabolically unhealthy obesity
OVX: ovariectomy
Preg: pregnenolone
PKA: protein kinase A
PLIN: perilipin
SAT: subcutaneous adipose tissue
SHBG: sex hormone binding globulin
StAR: steroidogenic acute regulatory protein
sWAT: subcutaneous white adipose tissue
T: testosterone
T2D: type 2 diabetes
TNFα: tumor necrosis factor alpha
VAT: visceral adipose tissue
vWAT: visceral white adipose tissue
WAT: white adipose tissue
WHI: Women’s Health Initiative
WHR: waist-to-hip ratio
WT: wild type.
